# Controlled viscous fingering in volatile fluid towards spontaneous evolution of ordered 3D patterns

**DOI:** 10.1038/s41598-023-35510-z

**Published:** 2023-06-30

**Authors:** Makrand A. Rakshe, Prasanna S. Gandhi

**Affiliations:** Suman Mashruwala Advanced Microengineering Laboratory, Department of Mechanical Engineering, Indian Institute of Technology Bombay, Powai, Mumbai, 400076 India

**Keywords:** Fluid dynamics, Mechanical engineering

## Abstract

Mimicking nature using artificial technologies has always been a quest/fascination of scientists and researchers of all eras. This paper characterizes viscous fingering instability-based, lithography-less, spontaneous, and scalable process towards fabrication of 3D patterns like nature-inspired honeycomb structures with ultra-high aspect ratio walls. Rich experimental characterization data on volatile polymer solution evolution in a uniport lifted Hele-Shaw cell (ULHSC) is represented on a non-dimensional phase plot. The plot with five orders of magnitude variation of non-dimensional numbers on each axis demarcates the regions of several newly observed phenomena: ‘No retention’, ‘Bridge breaking’, and ‘Wall formation’ with ‘stable’ and ‘unstable’ interface evolution. A new non-dimensional ratio of the velocity of evaporating static interface versus lifting velocity is proposed for the same. This phase plot along with physical insights into the phenomena observed, pave pathways for extending the method to multiport LHSC (MLHSC) to demonstrate multiwell honeycomb structures. The work thus establishes a solid foundation with valuable insights for scalable manufacturing of devices useful for application in biomedical and other domains.

Nature has been a significant source of inspiration for development of technologies^1–4^. Fractal-like geometries and patterns, observed ubiquitously in nature, have been mimicked in 2.5D geometries recently^5^ via a novel fluid shaping method exploiting viscous fingering instability in Hele-Shaw cell. Furthermore, a multiport lifted Hele-Shaw cell (MLHSC)^6^ has been proposed along with strategic locations of ports to obtain families of fractal-like and array patterns.

The physics behind the fluid shaping^5,6^ is based on exercising control over Saffman Taylor instability^7^,  also known as viscous fingering. It is an interfacial instability that occurs when a low viscous fluid is forced to flow into a high viscous fluid film, generating fascinating fractal-like patterns. Fluid flow in porous media^8,9^,  and fluid flow in a linear^7,10,11^ or radial^12^ Hele-Shaw cell experience this fingering instability^12–16^. A lifted Hele-Shaw cell (LHSC)^17–21^ is a modified version of a conventional Hele-Shaw cell. In LHSC, a viscous liquid droplet is first sandwiched and squeezed between two parallel plates to form a circular film and then the plates are separated after a small delay. Plate separation induces a parabolic pressure drop in the liquid film dragging the surrounding low viscous air into the film. The circular liquid film interface disintegrates randomly at multiple places because of Saffman Taylor instability, producing air fingers on the boundary. Upon complete separation, growth and shielding of these air fingers shape the fluid film into fractal-like patterns. This naturally scalable phenomenon of spontaneous reorganization of circular liquid film into branched-like patterns is the basis of the work in this paper.

Viscous fingering in LHSC has been studied widely in both experimental^19,20^ and theoretical domain^17,22^ using mainly Newtonian fluids. Nase et al.^20^ characterized force during viscous fingering, and Thamida et al.^19^ investigated tip splitting and shielding phenomena experimentally. Further, several modifications of LHSC plates have been proposed to probe their effect on interface evolution. Some examples are parallel grooves^23^,  circular grooves^24^,  and square lattice^25,26^ over the entire cell plate. In contrast, a recent work of Tanveer et al. proposed source pits^5^ and source holes^6^ (Uni and Multiple ports), as seeds for air fingers to initiate, at strategically planned locations to exercise strong control over otherwise random evolution of the liquid interface. Their ideas culminated in the development of recipes for systematic fabrication of multiscale, 2.5D fractal-like geometry and array patterns in MLHSC(Multiport LHSC) using the characterization of the evolution of interface in ULHSC (Uniport LHSC). Theoretical analysis and simulation of interface evolution in ULHSC^22^ offered deeper insights into this^6^ experimental work. Research in the interface instability in LHSC with non-Newtonian fluids is still evolving^5,6,27–31^and could not be found for volatile solutions to the best of our knowledge. However, static paint film drying^32^,  dewetting patterns in a drying liquid film^33^ and dewetting leading to fingering during evaporation of polymer film^34^ on a stationary surface have been researched.

In this paper, using a volatile polymer solution, we introduce a method to fabricate three dimensional, scalable, high aspect ratio structures via controlled stretching of fluid interfaces in the third dimension (direction of separation) during their evolution in U/M LHSC. Figure 1.a presents typical steps in the process, demonstrated for a case of evolution of volatile solution interfaces in ULHSC. The experiment begins with dispensing and squeezing a droplet of volatile solution on a cell plate with a sealed central hole (steps I and II). The seal is then removed, and the plates are separated (step III) to form the intermediate structure shown in step IV. Further separation, under specific settings, stretches the formation in the direction of separation to yield a 3D structure with a thin wall shown in step V. Due to the presence of a central hole, the evolution of inner interface, in addition to the outer interface, takes place towards pressure minima (midway between these interfaces). A stable (Fig. 1b) or unstable (Fig. 1c) evolution can commence depending on parameters. Under certain settings, with highly volatile solutions triggering the step V, 3D structures shown in Fig. 1d and e are accomplished. To obtain quantitative insights into this process and to facilitate further the fabrication of the desired 3D structures, we conduct a series of experiments and systematically characterize the results. Furthermore, we develop additional qualitative insights to extend the process further for accomplishing 3D array structures using MLHSC.

**Figure 1:**
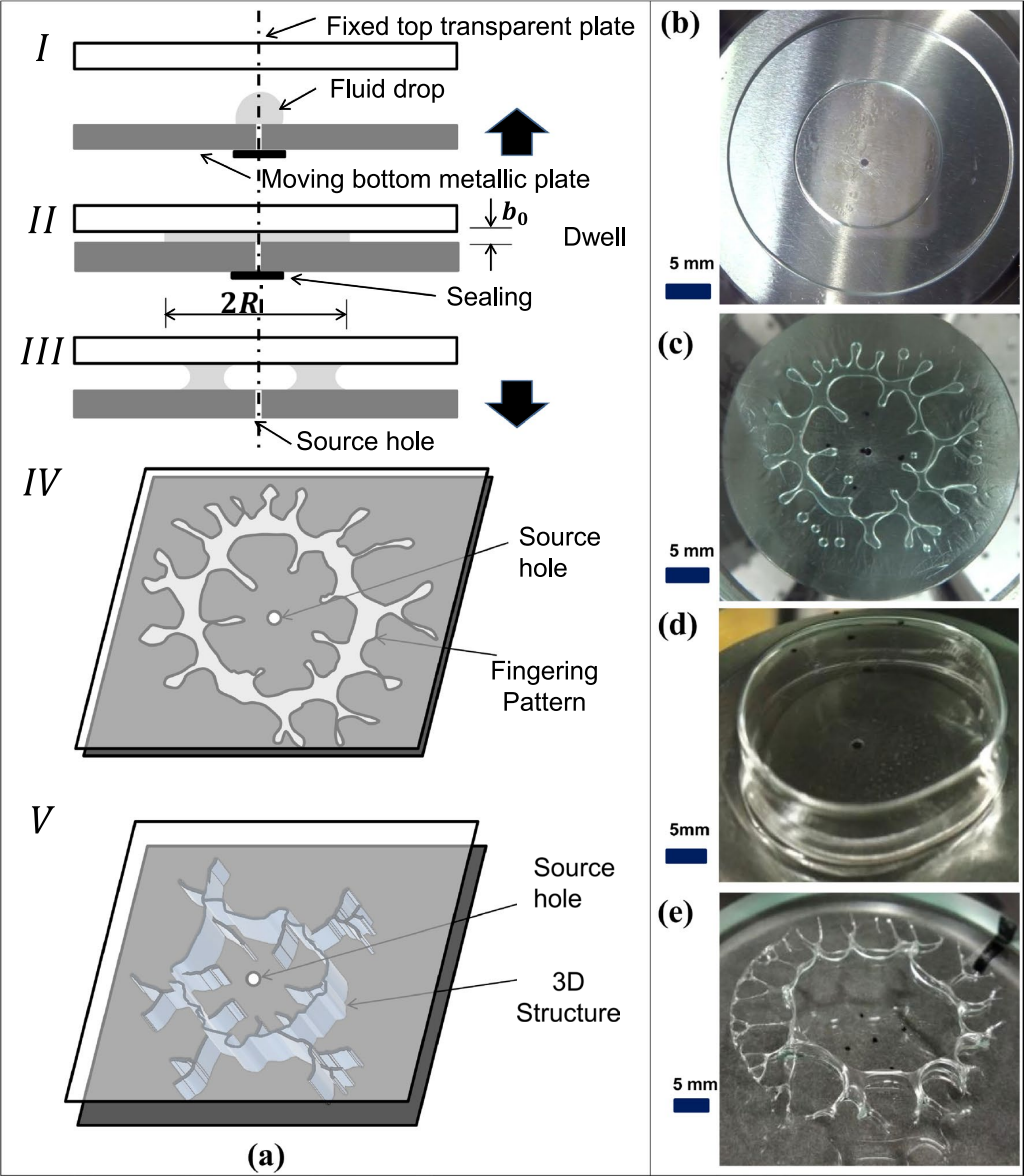
Schematic representation of the proposed method for fabrication of 3D structures and examples of evolved structures (**a**) Steps in the proposed process shown for Uni-port Lifted Hele-Shaw Cell (ULHSC). (**a.I**) Dispensing and squeezing of measured volume of a volatile solution droplet over the bottom plate with a sealed central hole. (**a.II**) Dwell for a few seconds to neutralize normal stresses in the squeezed fluid film. (**a.III**) Separation with unsealed hole (**a.IV**) Intermediate structure formation. (**a.V**) Final 3D structure formation by pinning and stretching of the interface along with solvent evaporation under certain settings. (**b**) Experimental image of step (**a.IV**) with stable evolution (**c**) Experimental image of step (**a.IV**) with unstable evolution (**d**) Experimental image of step (**a.V**): Hollow thin walled cylinder (**e**) Experimental image of step (**a.V**): 3D structure with parasitic fingers.

Experimental characterization begins with the validation of our experimental data with the previously published results in the regions where the effects of solvent evaporation are weak. A smooth deviation from these results is noticed when evaporation starts dominating. Two non-dimensional numbers influencing the phenomena are carefully identified for representing the experimental data on a phase map. Several new phenomena, indicating interesting fluid shaping and fabrication possibilities, are demarcated on this phase map with non-dimensional numbers spanning five orders of magnitudes on each axis. This map forms a solid foundation,

- for extension of this study for other volatile solutions and
- for providing rich information on parameter selection towards the fabrication of families of 3D structures.

A set of non-dimensional numbers indicate a region of particular interest on this map, with dominant volatility, where liquid-bridge stretching finally results in structures with thin solid walls with an ultra-high aspect ratio (UHAR) (thickness $\approx 10$ $\upmu$m, see supplementary Fig. 1, the height of wall/ wall thickness $\approx$ 350). Since Weber numbers for our experiments are generally low (of the order $10^{-7}$), inertia effects do not play a significant role in bridge stretching. Note also that in our case bridge has a complex 2D morphology unlike cylindrical counterparts studied previously in the literature^35^. The formation mechanism of the UHAR bridge and walls, in our case, is attributed to the effects of fast evaporation of the solvent (from the inner and outer surfaces of the bridge exposed to air) forming ultra high viscosity polymer chains on the surface of the bridge leading to very high local Ohnesorge number. A high Ohnesorge number is known to yield larger bridge stretching before rupturing in axisymmetric evolution during coaxial stretching^35^.

Gaining further qualitative physical insights into the process, the wall formation idea is extended to MLHSC^6^ with an appropriate choice of non-dimensional numbers to manufacture an array of square, hexagonal, or triangular shaped UHAR wells. The array of HAR micro-meso wells has been demonstrated to have several applications, including cell spheroid formation and drug screening^36^,  stem cell research, and tissue engineering^37^. In another possible application, multiple honeycombs (array of HAR hexagonal wells) can be stacked to yield 3D metamaterials^38^.

The proposed method of evaporation-induced fluid shaping to systematically synthesize families of 3D structures, including the HAR array of wells of different shapes, is simple yet elegant, robust, cost-effective, time-efficient, and scalable. These unparallel advantages of this fabrication process for such structures are reported for the first time to the best of our knowledge.

## Results

**Figure 2:**
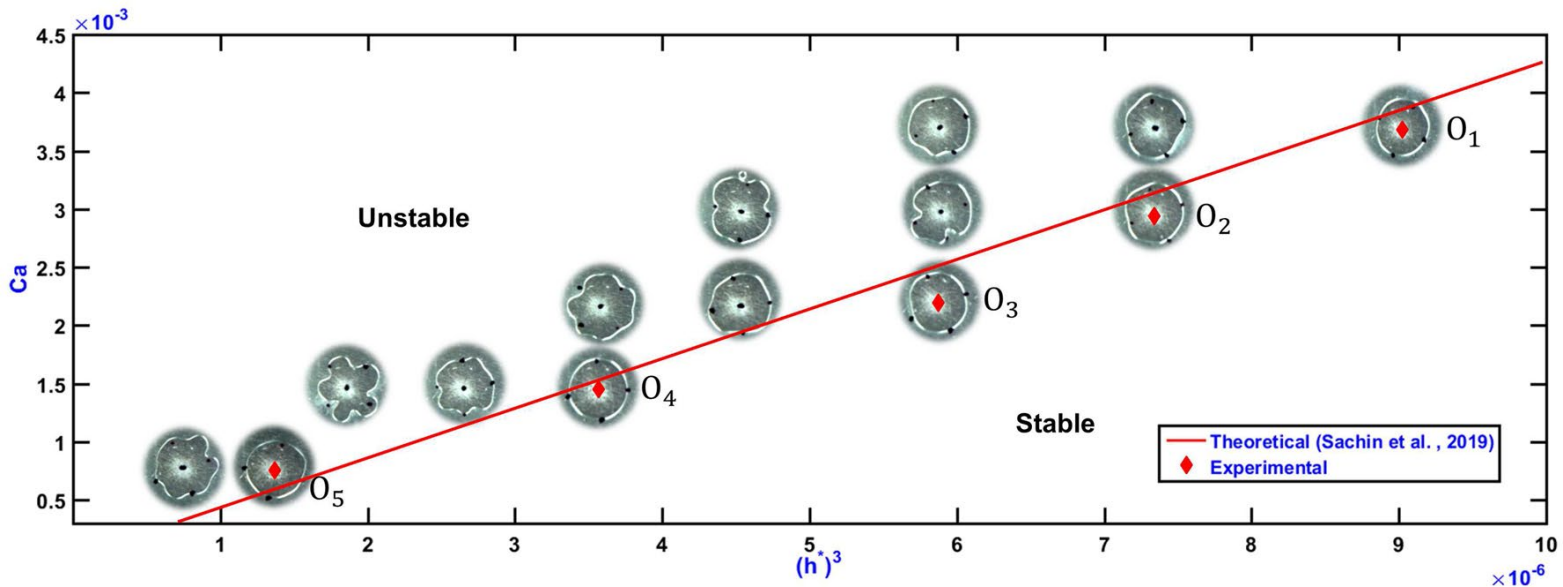
Stability map for the inner interface evolution of volatile polymer solution ‘$V_{c}$’ in a Uni-port LHSC at high Ca (order $10^{-3}$) Pictures show evolved inner interface of fluid film with white colored closed curve; the outer interface is not shown. Four black markings seen in each image at diameter 10 mm are used as a reference for observation of interface to conclude its stability. Here $h^{*}$ is given by $h^{*}=(b_{0}/R)$, where $b_{0}$ and *R* are initial fluid film thickness (range 175 $\upmu$m to 375 $\upmu$m) and radius (= 18 mm), respectively. Capillary number Ca on y-axis is Ca =$\upmu$v/$\sigma$, where $\upmu = 0.324\, \mathrm{Ns/m}^{2}$ and $\sigma = 0.02\, \mathrm{N/m}$ are dynamic viscosity and surface tension of the chloroform solution ‘$V_{c}$’ (solute- PS (Mw 192000)). v is the velocity of plate separation (range 46 $\upmu$m/s to 228 $\upmu$m/s). Theoretical results are based on linear stability analysis^22^.

### Theoretical versus experimental stability analysis in ULHSC

This section lays down the process of identifying the fingering instability transition point in ULHSC and compares the experimental stability phase plot (for the proposed volatile solution) with the previous theoretical results (based on linear stability analysis), especially in the regime where evaporation effects are ignorable.

Linear stability analysis of viscous fingering for Newtonian fluids in ULHSC^22^ identified key non-dimensional numbers governing stability as: Capillary number *Ca*, $h^* = b_0/R$ ($b_0$ is initial cell plate gap and *R* is squeezed film radius), and $\gamma = R/R_h$ ($R_h$ is the hole radius). The analytical expression for the dimensionless growth rate of the inner interface ($\omega ^{*}_{in}$) in terms of these parameters and wavenumber (n), is given as^22^

1 $$\begin{aligned} \omega _{in,i}^{*} = \left[ -\frac{1}{2}+ c_{1}^{*}\frac{h^{*^{3}}}{12}\frac{\gamma ^2}{Ca}\right] +\left[ \frac{n}{2}+\frac{h^{*^{3}}\gamma ^3}{12}\frac{(n^{3}-n)}{Ca}+c_{1}^{*}\frac{h^{*^{3}}}{12}\frac{\gamma ^2 n}{Ca}\right] F(\gamma ), \end{aligned}$$

where, $c_{1}^{*}$ and $F(\gamma )$ are formulated as,

2 $$\begin{aligned} F\left( \gamma \right) &= {} \dfrac{\left( 1+\gamma ^{2n}\right) }{\left( 1-\gamma ^{2n}\right) }, \end{aligned}$$

3 $$\begin{aligned} c_{1}^{*}&= {} c_{1}\dfrac{R_{out}^{0}}{\sigma } = \dfrac{1}{log \gamma }\left[ \left( 1+\gamma \right) -\dfrac{3 Ca}{h^{*^{3}}}\left( 1-\dfrac{1}{\gamma ^2}\right) \right] . \end{aligned}$$

Since we maintain $\gamma = 45$ constant in all our experiments, only *Ca* and $h^*$ would govern the transition from unstable to stable viscous fingering. For a given *Ca* and $h^*$ pair, theoretical transition is characterized by obtaining maximum value $\omega ^{*}_{in,max}$ of growth rate $\omega ^{*}_{in,i}$ over large range of wavenumbers n. $\omega ^{*}_{in,max} > 0$ signifies unstable evolution of the fluid interface and $\omega ^{*}_{in,max} < 0$ would indicate stable evolution. At a given *Ca*, this transition would happen as $h^*$ is varied. This transition from positive $\omega ^{*}_{in,max}$ to negative $\omega ^{*}_{in,max}$ has been reported^22^ to happen at a constant $Ca/(h^*){^{3}}$ . Hence phase map between *Ca* and $(h^{*})^{3}$ is considered for representing both experimental and theoretical results.

We carry out a series of experiments with volatile polymer solution ‘$V_{c}$’ (details given in the materials section) at various *Ca* and $h^*$ to obtain the stability graph. Specifically, at each constant *Ca* value, $h^*$ (or $b_0$) is gradually increased, and growth of the inner interface is observed, and a point where its destabilization ceases is marked as a transition point (see red markers in Fig. 2). Note that *Ca* values for all these experiments are chosen such that evaporation effects are ignorable. The experimental results are superimposed on previous theoretical results in Fig. 2 along with pictures indicating the transition. Reasonably close match indicated, as expected, that at higher *Ca*, the transition to instability is similar to that for a Newtonian fluid (because the evaporation effects are ignorable).

### Deviation due to evaporation

**Figure 3:**
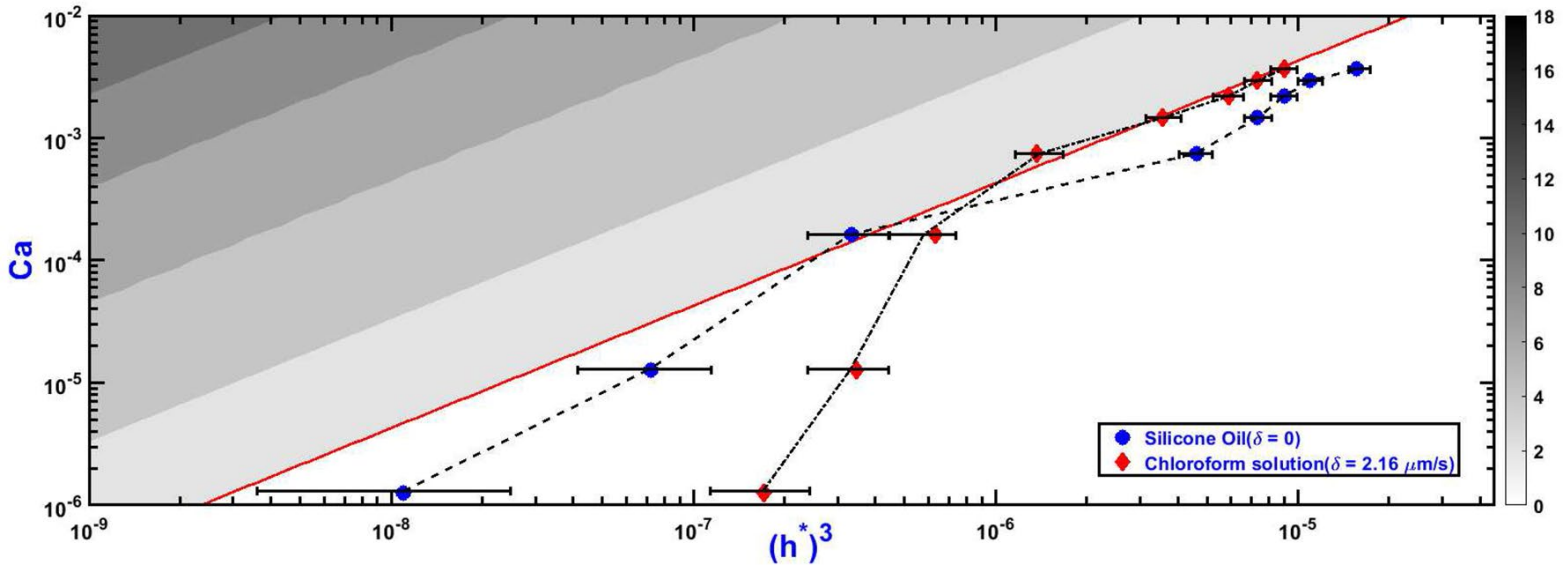
Experimental onset of stability curve for evaporative (‘$V_{c}$’) and non-evaporative (❛*So*’) fluid superimposed on theoretical results for Newtonion fluid. All curves represent onset of stability (transition from unstable to stable interface evolution zone) of the inner interface during evolution in the uni-port LHSC in the phase space of Ca and $(h^{*})^{3}$at a constant radius ratio ($\gamma = 45$). Different shades of the grey in unstable zone denote theoretical iso-contours of the maximum growth rate ($\omega ^{*}_{in,max}$, obtained as wavenumber n is varied) of the initial perturbations on the inner interface. The right side color bar shows the value of $\omega ^{*}_{in,max}$ corresponding to each shade. Red line indicates theoretical onset of stability. All theoretical results are based on linear stability theory^22^ (Eq. 1). Because of dominant evaporation at low *Ca* the onset of stability curve for ‘$V_{c}$’ deviates significantly from theoretical analysis with Newtonion fluid. All the experiments were conducted in the velocity range of 0.03–300 $\upmu$m/s and thickness range of 30–400 $\upmu$m. Dynamic viscosity and surface tension of fluids are: chloroform solution (‘$V_{c}$’) $\upmu = 0.324\, \mathrm{Ns/m}^{2}$ and $\sigma = 0.02\, \mathrm {N/m}$, and silicone oil ❛*So*’ $\upmu = 0.97 \,\mathrm {Ns/m}^{2}$ and $\sigma = 0.021\, \mathrm {N/m}$.

This section extends the results of stability analysis pertaining to our volatile polymer solution ‘$V_{c}$’ (details given in the materials section) for a wider range of *Ca* values (especially lower) where evaporation dominates. A procedure similar to the previous section is carefully followed to experimentally identify the onset of stability or the stability transition points. The results are now presented in Fig. 3 on a log-log scale along with previous theoretical results and experimental data points for a purely Newtonian fluid, silicone oil ‘*So*’, for clear comparison. In addition to the theoretical onset of stability where $\omega ^{*}_{in,max}$ flips the sign, we indicate iso-contours of $\omega ^{*}_{in,max}$ in the unstable region (where $\omega ^{*}_{in,max} > 0$) by using grayscale similar to that done in Sachin et al.^22^ Note that the viscosity of polymer solution ‘$V_{c}$’ would change due to evaporation effects during the process; we take initial viscosity for calculation of *Ca* in this plot.

Figure 3 shows a reasonable match, as expected, of the experimental stability curve with the theoretical predictions for Newtonian fluid ‘*So*’ and for polymer solution ‘$V_{c}$’ in high *Ca* region ( $> 10^{-4}$). However, the experimental stability curve for volatile polymer solution ‘$V_{c}$’ starts deviating significantly from the theoretical predictions at low Ca numbers ($10^{-6}$ to $10^{-4}$) where evaporation time scale starts competing with interface motion time scale. The deviation can be explained as follows: as the separation proceeds, the evaporative loss of solvent starts increasing the viscosity of the remaining solution. Recall that the onset of stability point at a given Ca is obtained by gradually increasing dimensionless initial fluid film thickness ($h^{*}$). For high apparent viscosity (as a result of evaporative solvent loss), higher $h^{*}$ is required for the onset of stability, thereby reducing the stability zone. Thus the stability curve for ‘$V_{c}$’ shifts into the theoretical stable zone, as observed in Fig. 3.

### New non-dimensional number *Ev*

During the execution of process steps mentioned in Fig. 1a, several new phenomena such as ‘bridge breaking’, ‘wall formation’ are observed, especially during steps IV to V, based on the relative strength of evaporation effects. To systematically represent/ classify these phenomena in a non-dimensional framework, a need for a suitable non-dimensional number corresponding to the effect of evaporation on the forced motion of the liquid interface (in our case in LHSC) is envisaged. After due consideration to several possibilities based on experimental observations, various dimensional quantities significant to the process of evolution of volatile solution interface in ULHSC were identified. Further, using Buckingham ‘pi’ theorem, a new non-dimensional number most significant to the process is proposed and termed as evaporative velocity ratio and designated as *Ev*. This number is defined as the ratio of interface velocity caused by pure evaporation ($\delta$) and lifting velocity (v) ($Ev = \delta / v$). We use this new non-dimensional number on the x-axis along with non-dimensional ratio $Ca/(h^*){^{3}}$ on the y-axis to generate a new plot that systematically characterizes significant effects of volatility in ULHSC observed as new phenomena, some of them not reported so far.

### Phase map of the various regions of fabrication

**Figure 4:**
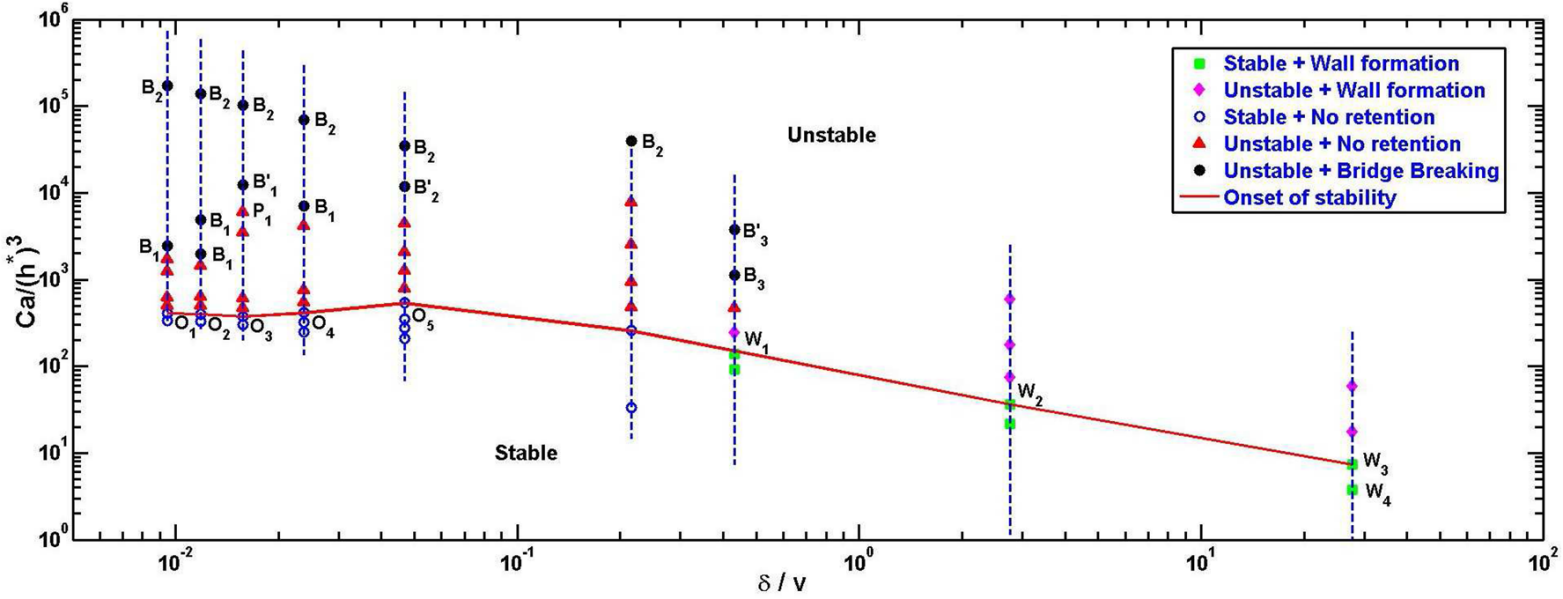
Phase map of the various regions of the fabrication in the space of evaporative velocity ratio ($\delta /v$) and modified Capillary number ($Ca/(h^*){^{3}}$): Vertical straight blue dashed lines are constant Ca lines; Here, each right side line has a low Ca value compared to left line. Along the constant Ca line aspect ratio increases from top to bottom. Different markers show the different regions of the fabrication. The Red line is the onset of stability; there is no viscous fingering (stable region) below this line. The horizontal nature of the onset of stability line (red line) shows the stability of the interface unaffected by volatility in a low $\delta /v$ (less than $10^{-1}$)) region. This red line deviates from the horizontal line in high $\delta /v$ (more than 0.05) region proves the fluid’s volatility also governs the interface stability. The stability region is decreasing as $\delta /v$ increasing. $\delta /v > 0.5$ always giving 3D (wall formation) structure. $O_1, O_2, O_3, O_4$ and $O_5$ denotes the experiment shown in the Fig. 2. $O_1$ and $P_1$ denotes experiment shown in the Fig. 5. $B'_1,B'_2$ and $B'_3$ denotes experiment shown in the Fig. 6. $W_1, W_2, W_3$ and $W_4$ denotes experiment shown in the Fig. 7.

According to linear stability theory^22^ for Newtonian fluids, at a constant radius ratio $\gamma$, $Ca/(h^*){^{3}}$ uniquely defines the onset of stability of the fluid-fluid interface in ULHSC (See red line of onset of stability in Fig. 3). The onset of stability in ULHSC for volatile polymer solution was presented partially in Fig. 2 and completely in Fig. 3 on a log scale to note its deviation from theoretical results because of the dominant effects of volatility. Using the proposed non-dimensional evaporative interface velocity ($Ev = \delta / v$) in the range up to 5 orders of magnitude and $Ca/(h^*){^{3}}$ in the range up to 5 orders of magnitude, we present a phase plot for fluid ‘$V_{c}$’ to characterize all our experimental observations in Fig. 4. The next section discusses stability results, to begin with.

#### Stable and unstable interface evolution

Each dashed vertical straight line in Fig. 4 represents a constant Capillary number line, and along each line, the aspect ratio $(h^{*})$ increases from top to bottom. Further, each vertical line on the right side has a lower Ca value than for the line on its left. At a given Capillary number higher the aspect ratio more stable the interface evolution (as evident from Figs. 2 and 3); thus, as we traverse along the constant Capillary number line from top to bottom, we hit a point of onset of stability captured by the red line connecting all such points (similar to the red line seen in Figs. 2 and 3). Theoretically^22^,  we understand that $Ca/(h^*){^{3}}$ is constant for a constant radius ratio for Newtonian fluids in ULHSC. We see in Fig. 4 that when the $\delta$ is small (ignorable evaporation effect), this red line is almost horizontal. The downward deviation of this line for higher $\delta$ is clearly in agreement with the observation in Fig. 3 of decreasing stable zone with the increase in evaporation.

**Figure 5:**
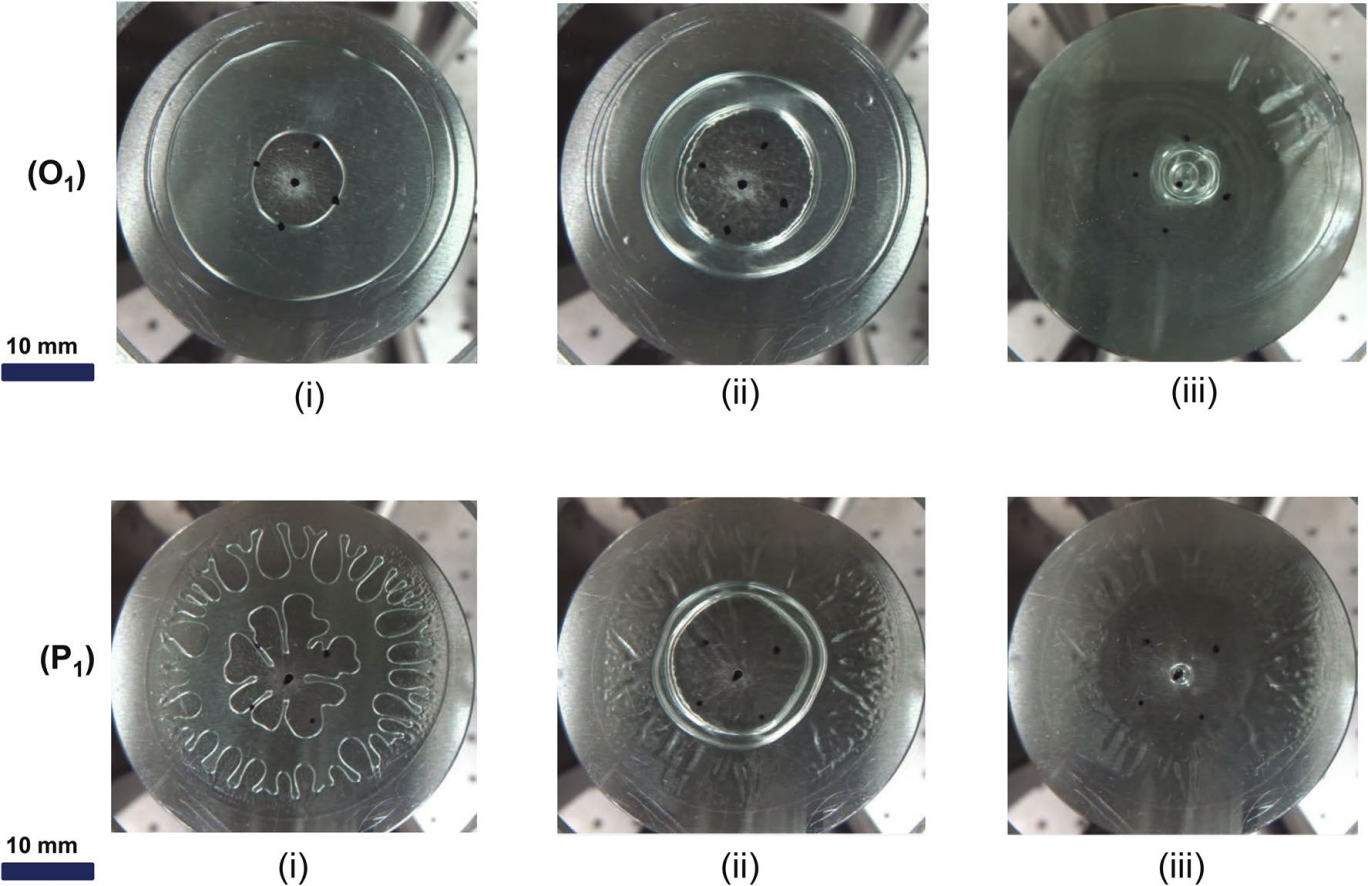
Experimental images of the No Retention region: Experiment $O_{1}$) This experiment (See Supplementary Video 1) was carried out in a stable region; hence separation starts with the stable evolution of the inner interface (image i). Initially, radius of the inner interface grows (image i to ii) with time due to the non-monotonic pressure profile in the fluid domain. Once dominance of the surface tension at the interfaces increases, the fluid domain’s pressure profile becomes monotonic; hence, further plate separation radius of both the interfaces starts shrinking (images ii to iii). This experiment is shown on the Fig. 4 by $O_1$. Experimental parameters in this experiment are v= 228 $\upmu$m/s and $b_{0}$=375 $\upmu$m. Experiment $P_{1}$) This experiment was carried out in an unstable region; hence separation starts with viscous fingering (image i). For further separation, first parasitic fingers get retracted into the fluid domain due to non-monotonic pressure profile and form a stable ring (image i to ii), and then same as the previous ring gets retracted (image ii to iii). This experiment is shown on the Fig. 4 by $P_{1}$. Experimental parameters in this experiment are v= 137 $\upmu$m/s and $b_{0}$ = 125 $\upmu$m.

#### No retention region

This region is observed (see Fig. 4) where $Ca/(h^*){^{3}}$ is upto $10^3$ or $10^4$ and *Ev* is upto 0.5. In this region, the evaporation effects are weak or ignorable; hence, the interface evolution is close to Newtonian fluid’s. The hallmark of this evolution, whether stable or unstable, is that the evolved interface shapes are not retained. Thus this region is not suitable for the proposed fabrication purpose but is discussed for the sake of completeness.

Closer observation revealed that the evolution of outer and inner fluid interfaces in this region is qualitatively similar to that discussed in^22^ with theoretical and numerical analysis for the case of Newtonian fluid evolution in ULHSC. We briefly discuss here two example cases of evolution (points $O_{1}$ and $P_{1}$ in Fig. 4) that demonstrate representative observation of transition in the direction of motion of the inner interface stable $O_{1}$ and unstable $P_{1}$ evolution.

Key snapshots in the evolution of the fluid interfaces as the LHSC plates are separated are shown in Fig. 5 for two cases $O_{1}$ and $P_{1}$. Initially, the inner interface grows and the outer interface shrinks (image i to ii) with time (with stable evolution in $O_{1}$ and unstable evolution followed by retraction in $P_{1}$), and with further plate separation both interfaces shrink (image ii to iii); thus no fluid structure is retained. This peculiar behavior is attributed to changing pressure profiles from non-monotonic (for i) to monotonic (for ii and iii) with corresponding shift in pressure minima at the center (midway between outer and inner boundaries) to pressure minima at the inner boundary, respectively. Initially, pressure at both boundaries is atmospheric, and pressure minima is midway between the inner and outer boundaries as the separation begins. The shift of pressure minima from the center (midway between boundaries) to the inner boundary happens due to dynamic change of $h_d^*$ (local gap (as plates separate) vs. R ratio) and $\gamma _d$ (local ratio of outer to inner radius) which is evident from qualitatively similar theoretical plots presented in Figs. 18 and 19 in Sachin et al.^22^ These plots are reproduced in the supplementary Fig. 8 and 9 with a more detailed explanation.

#### Bridge breaking before total retraction

**Figure 6:**
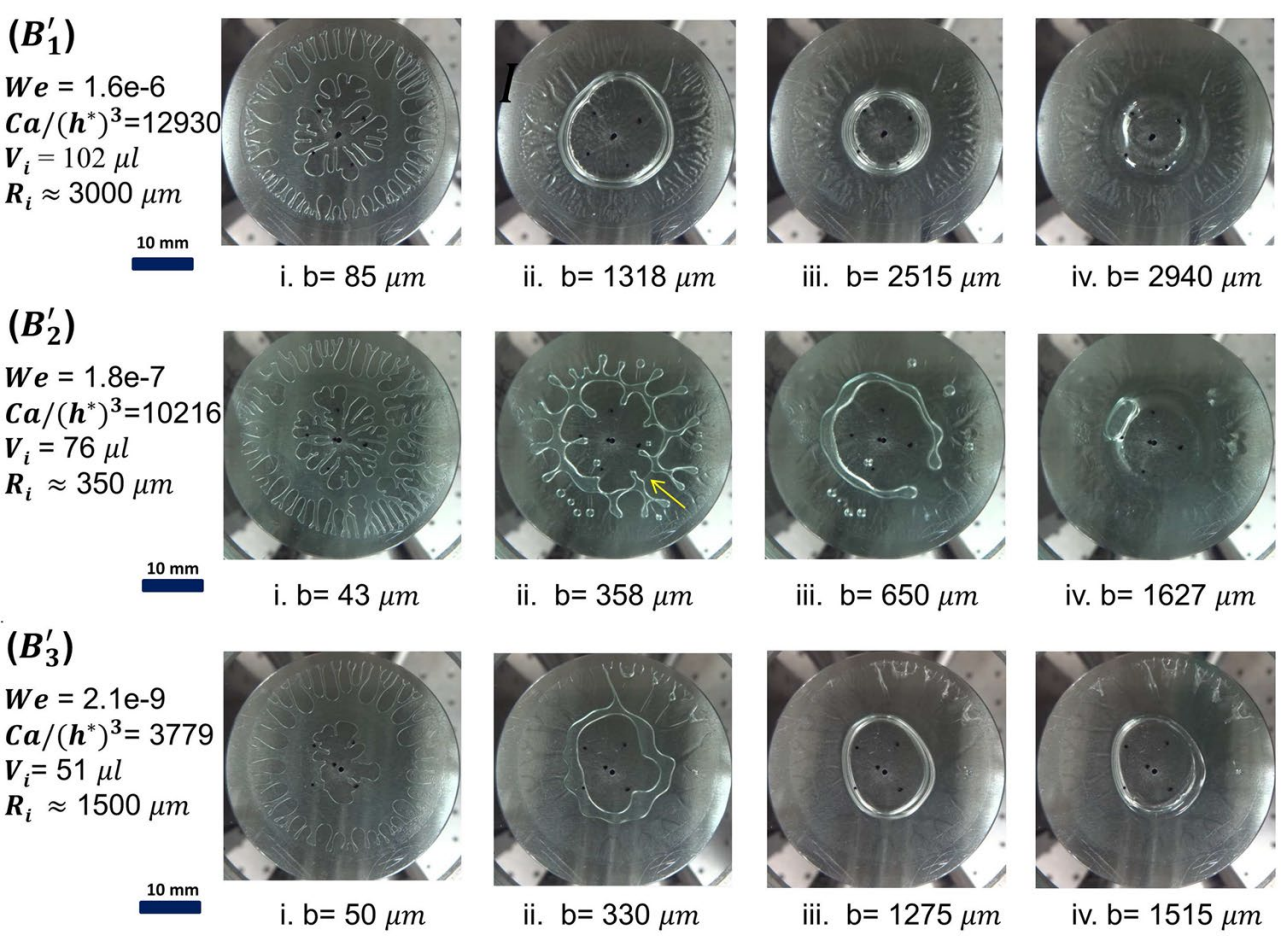
Bridge breaking before total retraction: $B'_{1}$ (See Supplementary Video 2), $B'_{2}$ (See Supplementary Video 3) and $B'_{3}$ are representative experiments. Weber numbers *We*, volumes $V_i$, bridge rupture distance $R_i$ and $Ca/(h^*){^{3}}$ are mentioned for each case. ‘b’ denotes the instantaneous gap between two plates and panels i-iv display the image of interface evolution at each of the b mentioned.

Bridge breaking zone is characterized by higher values of $Ca/(h^*){^{3}}$ (above the ‘no retention’ zone having relatively smaller $h^*$) and lower *Ev* as seen by all $B_i$ points marked in Fig. 4. In this region also, as mentioned earlier, the polymer solution behavior is close to Newtonian because the evaporation effects are relatively weak. The initial fluid evolution proceeds similar to that in ‘no retention’ zone; however, because the total volume of fluid is relatively small (small $h^*$) in these cases as compared to that in ‘no retention’ zone (on the same vertical dashed line), the bridge breaks instead of getting completely retracted. In bridge breaking cases, evolved interface shapes are partly retained till the bridge breaking occurs. Thus this region is also not suitable for the proposed 3D shaping; however, the insights developed in the study could be useful.

The bridge breaking zone shows three distinct ways the phenomena occur and is marked by $B_{1}$, $B_{2}$, and $B_{3}$ cases. Representative cases $B_1'$, $B_2'$, $B_3'$ are chosen for brief discussion here, and the snapshots of evolution (with successive plate separation) in each of these cases are presented in Fig. 6. All these cases begin with fingering instability as expected and are observed in the image in panel i in each case. Cases $B_1'$ and $B_3'$ progress in a similar way by reorganization of liquid into a ring and breaking of the ring, the only difference being the twice larger rupture distance in $B_1'$. In contrast, in case $B_2'$ partial bridge breaking starts much earlier and affects subsequent fluid reorganization as plate separation continues. To develop more physical insight into these observations, we borrow relevant insights from the previous literature^35^ in the following paragraph.

Experimental and numerical investigation in axisymmetric bridge breaking (due to coaxial stretching) of the filled cylindrical volume of Newtonian fluid^35^ reveals that the bridge rupture distance increases with increasing *We* and *Oh*. Here *We* ($We=\rho l_1 v^{2}/\sigma$) is Weber number (competition between inertial force and surface tension force), and *Oh* ($\upmu /(\rho l_1 \sigma )^{1/2}$) is Ohnesorge number (competition between viscous force and surface tension force). *Oh*, in our bridge breaking case, under discussion, can be considered constant ($\sim 1.75$) for all cases since the viscosity and surface tension are not changing in this zone because of weak evaporation. Furthermore, the volume of the liquid bridge in our case varies since the initial volume of fluid dispensed changes with $h^{*}$, and the bridge shape is governed by fingering instability. Thus interplay between *We*, $Ca/(h^*){^{3}}$, and the volume of liquid is used to explain varying bridge rupture distances ($R_i$ mentioned in Fig. 6) in cases $B_1'$, $B_2'$, $B_3'$.

As mentioned earlier, cases $B_1'$ and $B_3'$ show interface progression in a similar way, although the strength of fingering instability is more in $B_1'$ than in $B_3'$ indicated by respective $Ca/(h^*){^{3}}$ numbers and also evident in image panel i of Fig. 6; The larger bridge rupture distance in case of $B_1'$ is apparent because of twice higher volume than in case $B_3'$ and also 3 orders of magnitude higher *We*^35^. For case $B_2'$ fingering strength is almost similar to that in $B_1'$, however volume is much lesser. Therefore although *We* for this case is only one order of magnitude lesser than that of $B_1'$, the bridge ruptures very early, partially, where inner and outer boundaries are closest. This disturbance affects subsequent fluid reorganization as the separation continues and prevents fluid ring formation.

We will see in the next section that *Oh* becomes more important for wall formation with dominant evaporation effects.

**Figure 7:**
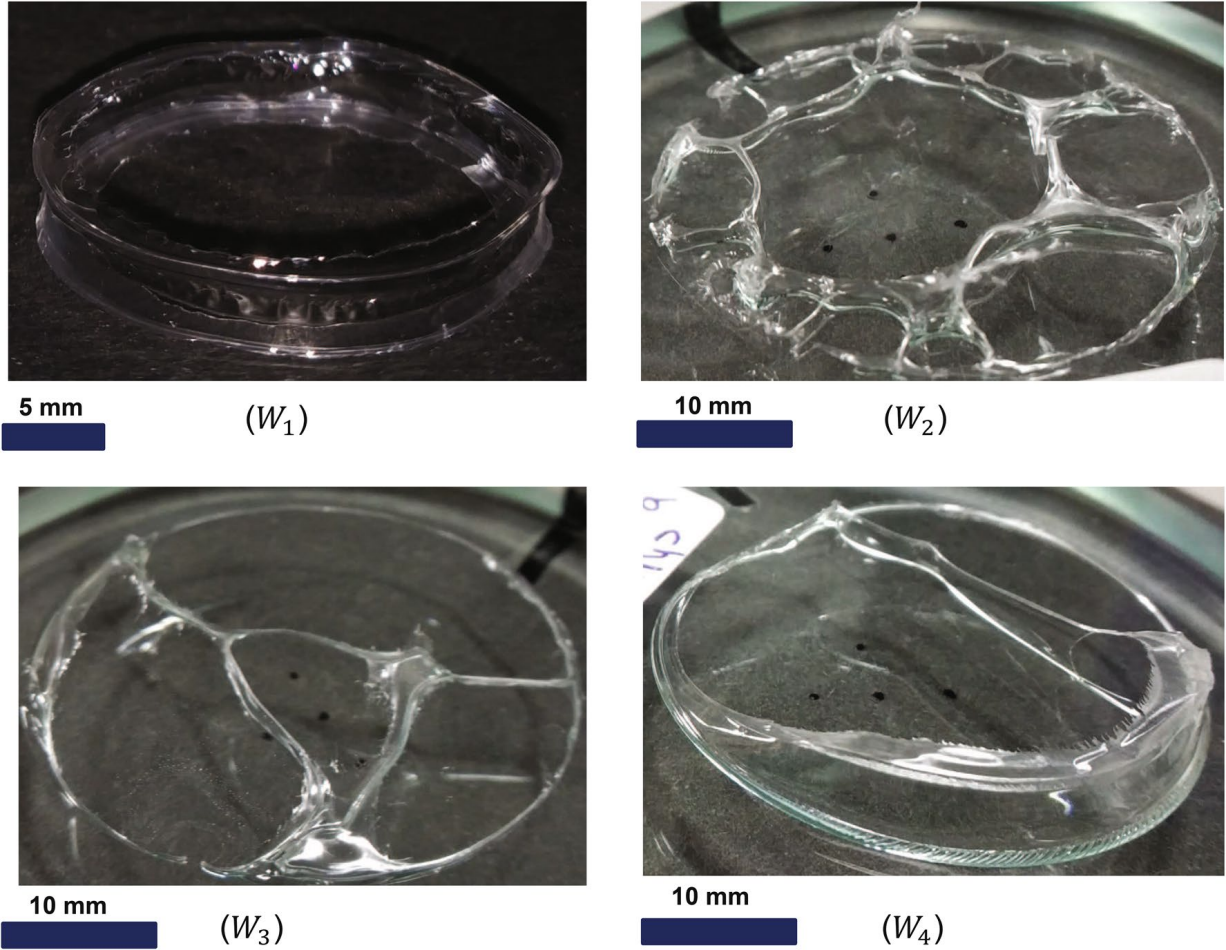
Wall formation $W_{1}$**)** Hollow cylindrical structure fabricated on Uni-port lifted Hele-Shaw cell. It is the same structure shown in Fig. 1. d released from the cell plates. This experiment was denoted by $W_{1}$ in Fig. 4. Experimental parameter in $W_{1}$ are v = 5 $\upmu$m/s, Ca = 0.000081, $Ca/(h^*){^{3}}$= 242, $\delta /v$ = 0.43, and fluid volume = 127 $\upmu$l. Wall thickness $\approx$ 10 $\upmu$m and height of the structure/wall thickness $\approx$ 350. $W_{2}$) Hollow 3D structure with parasitic fingers. This experiment was denoted by $W_{2}$ in Fig. 4. $W_{3},W_{4}$) In this experiment $\delta /v > 10$ , We observe the pinning of the interface here. Pinning of the outer interface inhibits the viscous fingering. These experiments denoted by $W_{3}$ and $W_{4}$ in Fig. 4.

#### 3D wall formation

In regions of low $\delta /v$ discussed so far, fluid shaping is followed eventually by complete retraction, 3D structures are not retained in these cases. As the dominance of $\delta$ over *v* increases (signifying dominant evaporation), dynamically changing viscosity and interface pinning effects give rise to an interesting phenomenon of wall formation, not reported so far. We observe the wall formation region beyond $Ev > 0.43$. This is the most relevant region for the fabrication of structures stretched in the third dimension. Furthermore, the highest control over the shape of the structures is obtained in the stable part of this zone (below the onset of stability line in Fig. 4).

Different stages of evolution leading finally to wall formation are classified as follows:

**Stage I: Saffman–Taylor instability** As the plate separation begins, the fluid interface evolves as stable or unstable depending on the choice of $Ca/(h^*){^{3}}$ and $\delta /v$ corresponding to a point either above or below to the stability line in Fig. 4.

**Stage II: Pinning of the interfaces** Evaporation leading to contact line pinning has been studied in the literature^39,40^ and is well known as the “coffee stain phenomenon” or “coffee ring effect”. This phenomenon was first reported by Deegan et al.^39^ A similar phenomenon, depending on surface energy, occurs in the process of drying polymer solution, which is explained by C. Poulard^40^. Poulard explained that outward Marangoni flow is generated in the drying process of polymer solution when the polymer (in our case polystyrene) has more surface energy than the solvent (in our case chloroform). The collection of more polymer at the boundary, due to this flow, leads to the pinning of the moving interface. In our case, when $\delta /v$ is more, i.e., in the evaporation dominant regime, since the velocity of interface motion due to plate separation is small, the coffee stain phenomenon dominates, leading to pinning of the interface.

In experiment $W_{1}$, the initial value of the *Ev* is 0.43 (see Fig. 4), which means initially, interface motion due to plate separation is dominating over the motion of the interface due to evaporation. Thus we observe the smooth motion of both inner and outer interfaces without pinning till the displaced fluid forms a stable ring. However, beyond this point, due to slower interface motion in addition to continuous evaporation, the coffee stain phenomenon dominates, and the interface gets pinned, which prevents further motion of the interface even if the plate separation continues (See Fig. 7). As $\delta$ dominance over *v* is further increased ($\delta /v >$ 1 in case of $W_{2}$, and $> 10$ in case of $W_{3}$ and $W_{4}$), the pinning of the outer interface of the squeezed fluid film to the cell plates takes place even before the plate separation begins, which hinders the viscous fingering from happening (partially in $W_{2}$ and completely in $W_{3}$ and $W_{4}$) at the outer interface. As a result, the outer pinned interface is locally punctured (See Supplementary Fig. 3) during the cell plate separation. Upon further separation, fluid shaping happens due to air suction from puncture points and from the middle source hole (See Fig. 7.$W_{2}$, $W_{3}$ and $W_{4}$) in uncontrolled fashion.

**Figure 8:**
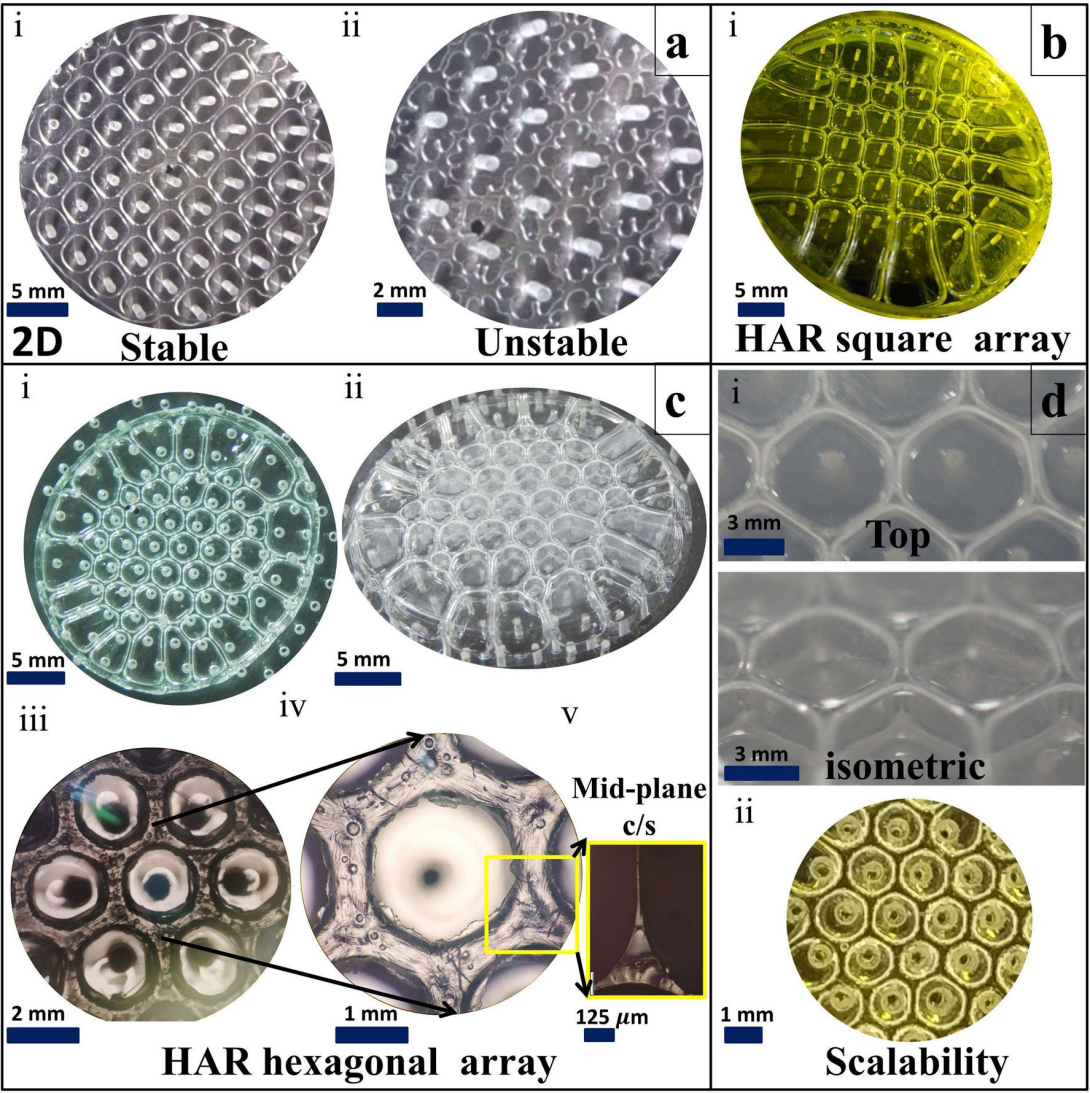
Fluid shaping of volatile polymer solution (120% w/v chloroform solution) over Multi-port Lifted Hele-Shaw cell (MLHSC) (**a**) Experimental image of the air finger evolution in MLHSC (i) Stable evolution of square array (ii) Unstable evolution of square array (**b**) i. HAR square array (**c**) HAR hexagonal array height = 2500 micron (equal to final separation of the cell plates), height/wall thickness = 250 (i) Top view (ii) Isometric view (iii) Microscopic top view (iv) Zoomed top view of single hexagonal well (v) Microscopic image of the mid-plane (along the height of the structure) cross-section of the yellow highlighted part. **d)** Scalability of the hexagonal array fabrication (i) Large hexagonal mesh, each side of hexagone = 3.5 to 4 mm (ii) Small hexagonal mesh, each side of hexagone = 1 mm. (In case of the (**b**).i and (**d**).ii, yellow light is focused on the structure to capture the edges of the structure sharply).

**Stage III: Stretching and evaporation** Pinning of the fluid film interface, in the previous stage, inhibits the radial motion of the interface and leads to the formation of structures (see Fig. 7 and cases $W_1-W_4$ in Fig. 4) in the following way: After pinning of the interface, continued dominance of evaporation and formation of polymer chains, especially on the inner and outer surfaces of the bridge, leads to sharp local (on surface) increase of Ohnesorge number *Oh*. An increase of *Oh* is known to sustain more stretching of the interface^35^. In our case, after plate separation stops, the stretched bridge is retained as a high aspect ratio (HAR) wall by completing the evaporation of the solvent in the drying process. Eventually, hollow cylindrical structures with (Fig. 7$W_2$) and without (Fig. 7$W_1$) parasitic HAR walls are retained. In similar way, structures $W_3$ and $W_4$ evolve.

### Fabrication of the high aspect ratio (HAR) well-ordered scalable patterns on MLHSC

Insights developed so far indicate that the stable wall formation regime in Fig. 4 with *Ev* in range around 0.3–0.6 would be ideal for extending the method to multiport LHSC. In the case of a non-volatile solution, multiport LHSC is known to give a more stable evolution of the air finger as compared to a single source port LHSC because air finger evolution from each port/hole adds a shielding effect on the adjacent air finger growth^6^ and this stabilizing effect keeps increasing with the decreasing source-holes pitch^41^. However, while using a volatile solution in MLHSC, there is an additional consideration: the stretched fluid film interface would form multiple pockets in which volatile solvent vapors would get trapped and slow down/ prevent further evaporation. This shielding of evaporation (which otherwise was absent in single port LHSC) will prevent retained wall formation in the same regime for MLHSC. It follows that we need to use a polymer solution with relatively less solvent content (in other words, higher polymer concentration). Considering this qualitative insight, we extend ideas developed in the paper for a single port to MLHSC using a high concentration ( 120% w/v) polymer solution. The results of the hexagonal and square well array fabrication with high aspect ratio walls are presented in Fig. 8. For example, the hexagonal arrayed multiwell pattern shown in Fig. 8.c has a high aspect ratio ($\approx 250$) (here, aspect ratio is the ratio of the height of the hexagon well to the wall thickness (at mid-plane (see Supplementary Fig. 4)) of the hexagonal wells although the cross-section is tapering towards mid-plane from both sides.).

We also observe that the process is suitable to fabricate such patterns over a large area as long as a favorable range of non-dimensional numbers, by taking cues from Fig. 4, are maintained. Figure 8d.i and d.ii present a similar structure formation at two different sizes. Since the process has been characterized in non-dimensional numbers, one can see similar possibilities at multiple scales.

## Conclusion and discussion

This paper experimentally demonstrated a robust and repeatable process of fluid shaping of volatile polymer solution in the uni-port LHSC by controlling Saffman-Taylor instability. Non-dimensional characterization of the volatile polymer solution in uni-port lifted Hele-Shaw cell is presented using two non-dimensional phase plots, stability graph ($(h^*){^{3}}$ vs. Ca) and phase map demarcating various regions of evolution ($Ev = \delta /v$ vs. $Ca/(h^*){^{3}}$). A new non-dimensional number *Ev*, evaporative velocity ratio, was introduced, for the first time to the best of our knowledge, for the same. The stability graph presented a comparative analysis of theoretical and experimental fingering instability results and captured their gradual deviation, signifying increasing effects of volatility. The $Ev = \delta /v$ vs. $Ca/(h^*){^{3}}$ phase map provided a rich understanding about how a volatile polymer solution interfaces would evolve in ULHSC for varying characteristics non-dimensional numbers. The most important region for development of 3D stretched structures was identified to be the region of ‘wall formation’ marked by *Ev* in range 0.3-0.6 where fluid is initially able to flow in 2D, but then would stretch in 3rd direction after interface pinning. Moreover qualitative understanding of evaporative shielding effects was considered for further extension of the method to mulit-port LHSC.

Tuning the experimental parameter based on the cues taken from non-dimensional graph along with additional qualitative understanding, enabled the shaping of the volatile polymer solution into a family of scalable 3D patterns with high aspect ratios in MLHSC. This fabrication process is time efficient due to spontaneous fluid shaping and evaporation driven self curing. In addition to this, the proposed way of fabrication is cost-effective and scalable as well. Such a process, along with non-dimensional characterization, has been proposed for the first time to the best of our knowledge and can form a solid foundation for future developments of both academic and application interest. The structures fabricated using this process (see for example, Fig. 8) could find applications in the domains of cell spheroid formation, stem cell research, drug screening, and tissue engineering. 3D array structure similar to a honeycomb may find use toward metamaterial development.

## Methods

### Experimental setups

Uni-port lifted Hele-Shaw cell setup consists of two cell plates (see the setup picture in Supplementary Fig. 5). The top glass plate allows visualization of the process, and the bottom SS 410 plate has a single central hole (uniport) in ULHSC experiments. In the MLHSC case, acrylic plate with multiple ports precision drilled at strategically planned locations is used in place of uniport metal plate. The LHSC experiments are sensitive to a relative tilt in the plates; hence universal joint-like self-paralleling mechanism is incorporated in the setup to achieve a parallelity within 5 microns over a diameter of 50 mm. In this setup, the top plate is fixed while the bottom plate mounted on the motorized translation stage (MTS, from Holmarc) actuated by a stepper motor via the dSPACE data acquisition system. Codes in Matlab Simulink$^{TM}$ were developed to accurately control squeezing and separation speeds and dwell time in between. A magnetic encoder (Renishaw, resolution 1 micron) is mounted on the setup to get the position of the bottom plate. Sealing and unsealing of the single or multiple ports on the bottom plate is done manually during dwell period. A thin layer of silicon rubber is sandwiched between a strong magnet and the plate to ensure proper sealing.

### Fluid preparation

Polystyrene (Mw 192000, Sigma Aldrich) $30\%$ w/v is dissolved into organic solvent chloroform to get volatile polymer solution $V_{c}$. The dissolving process is accelerated by continuously stirring the solution with a magnetic stirrer (IKA, RET CV W S000). Stirring of polystyrene and the solvent is carried out inside a glass bottle with a rubber seal for 50 minutes. Fluid properties details are in the supplementary files (see Supplementary Figs. 6 and 7). The procedure for calculating the evaporation rate in m/s is mentioned in the supplementary file. The fluid used in MLHSC for HAR structure fabrication is a chloroform solution having a polystyrene concentration of 120% w/v.

### Design of experiment

The following procedure is followed for LHSC experiment,

**Step 1**: Initially measured volume of fluid drop put on the bottom plate with the help of syringe (least count of syringe $\pm 10 \upmu \textrm{l}$). The ports on the bottom plate are sealed while squeezing. Using encoder position feedback, fluid is squeezed to the required thickness with the tolerance of $\pm 1 \upmu \textrm{m}$.
**Step 2**: Squeezing is followed by a few seconds of dwell to neutralize normal stress in the fluid.
**Step 3**: The hole/port on the bottom plate is unsealed, and plates are separated at the desired constant velocity.

The experimental procedure is carried out on a mechatronic setup (developed at Suman Mashruwala Advanced Microengineering Laboratory, IIT Bombay) in a sequence programmatically using the dSPACE data acquisition card, and codes developed in-house. Squeezing time (12–15 sec.) and radius (= 18 mm) for each experiment are maintained identical to ensure the evaporative loss of solvent during squeezing, although very tiny, is identical for every experiment. The experimental process is captured into a camera (NIKON, DSC-WX220) at 30 fps.

As seen in Fig. 2, we initially conducted experiments for five Capillary numbers (Ca), by varying the separation velocity. For each Ca, we conducted an experiment for various aspect ratios $h^*$ by gradually increasing initial fluid film thickness in steps of 25 micron and by keeping the radius of the squeeze constant. This process is repeated until we get finger-free evolution of the inner interface. The same procedure is carried out for the remaining lower-order Capillary number, and a stable aspect ratio is experimentally determined (Fig. 3). The onset of stability experiment is repeated three times for each Capillary number. Tolerance on the values would be attributed to limit on the steps for initial fluid film thickness, and manual observation of finger-free evolution.

## Supplementary Information

Supplementary Information 1.

Supplementary Information 2.

Supplementary Information 3.

Supplementary Information 4.

## Data Availability

All data generated or analysed during this study are included in this published article and its supplementary information files.

## References

[1] Bar-Cohen Y (2012). Nature as a model for mimicking and inspiration of new technologies. Int. J. Aeronaut. Space Sci..

[2] Bar-Cohen Y (2005). Biomimetics: mimicking and inspired-by biology. Smart Struct. Mater. 2005 Electroact. Polym. Actuators Dev. (EAPAD).

[3] Cui F (2018). Electrospinning: A versatile strategy for mimicking natural creatures. Compos. Commun..

[4] Li M, Li C, Blackman BR, Eduardo S (2021). Mimicking nature to control bio-material surface wetting and adhesion. Int. Mater. Rev..

[5] Ul Islam T, Gandhi PS (2016). Fabrication of multscale fractal-like structures by controlling fluid interface instability. Sci. Rep..

[6] Ul Islam T, Gandhi PS (2017). Viscous fingering in multiport Hele Shaw cell for controlled shaping of fluids. Sci. Rep..

[7] Saffman PG, Taylor G (1958). The penetration of a fluid into a porous medium or Hele-Shaw cell containing a more viscous liquid. Proc. R. Soc. Lond. A Math. Phys. Eng. Sci..

[8] Homsy GM (1987). Viscous fingering in porous media. Annu. Rev. Fluid Mech..

[9] Chen JD, Wilkinson D (1985). Pore-scale viscous fingering in porous media. Phys. Rev. Lett..

[10] Nittmann J (1986). Fractal viscous fingering: Experiments and models. Phys. A.

[11] Al-Housseiny TT, Tsai PA, Stone HA (2012). Control of interfacial instabilities using flow geometry. Nat. Phys..

[12] Paterson L (1981). Radial fingering in a Hele Shaw cell. J. Fluid Mech..

[13] Tryggvason G, Aref H (1983). Numerical experiments on Hele Shaw flow with a sharp interface. J. Fluid Mech..

[14] Tabeling P, Zocchi G, Libchaber A (1987). An experimental study of the Saffman-Taylor instability. J. Fluid Mech..

[15] Tryggvason G, Aref H (1985). Finger-interaction mechanisms in stratified Hele-Shaw flow. J. Fluid Mech..

[16] Chen JD (1989). Growth of radial viscous fingers in a Hele-Shaw cell. J. Fluid Mech..

[17] Shelley MJ, Tian FR, Wlodarski K (1997). Hele-Shaw flow and pattern formation in a time-dependent gap. Nonlinearity.

[18] Lindner A, Derks D, Shelley MJ (2005). Stretch flow of thin layers of Newtonian liquids: Fingering patterns and lifting forces. Phys. Fluids.

[19] Thamida S, Takhistov P, Chang H-C (2001). Fractal dewetting of a viscous adhesive film between separating parallel plates. Phys. Fluids.

[20] Nase J, Derks D, Lindner A (2011). Dynamic evolution of fingering patterns in a lifted Hele-Shaw cell. Int. Commun. Heat Mass Transf..

[21] Ul Islam T, Gandhi PS (2017). Spontaneous fabrication of three-dimensional multiscale fractal structures using Hele-Shaw cell. J. Manuf. Sci. Eng..

[22] Kanhurkar SD, Patankar V, Ul Islam T, Gandhi PS, Bhattacharya A (2019). Stability of viscous fingering in lifted Hele-Shaw cells with a hole. Phys. Rev. Fluids.

[23] Horváth V, Vicsek T, Kertész J (1987). Viscous fingering with imposed uniaxial anisotropy. Phys. Rev. A.

[24] Sinha S, Kabiraj SK, Dutta T, Tarafdar S (2003). Radially interrupted viscous fingers in a lifting hele-shaw cell. Eur. Phys. J. B-Condens. Matter Complex Syst..

[25] Ben-Jacob E (1985). Experimental demonstration of the role of anisotropy in interfacial pattern formation. Phys. Rev. Lett..

[26] Banpurkar AG, Limaye AV, Ogale SB (2000). Occurrence of coexisting dendrite morphologies: Immiscible fluid displacement in an anisotropic radial Hele-Shaw cell under a high flow rate regime. Phys. Rev. E.

[27] Fast P, Kondic L, Shelley MJ, Palffy-Muhoray P (2001). Pattern formation in non-Newtonian Hele-Shaw flow. Phys. Fluids.

[28] Fontana JV, Miranda JA (2013). Finger competition in lifting hele-shaw flows with a yield stress fluid. Phys. Rev. E.

[29] Fontana JV, Dias EO, Miranda JA (2014). Controlling and minimizing fingering instabilities in non-Newtonian fluids. Phys. Rev. E.

[30] Roy S, Tarafdar S (1996). Patterns in the variable Hele-Shaw cell for different viscosity ratios: Similarity to river network geometry. Phys. Rev. E.

[31] Kabiraj SK, Tarafdar S (2003). Finger velocities in the lifting Hele-Shaw cell. Phys. A.

[32] Goehring L, Li J, Kiatkirakajorn PC (2017). Drying paint: from micro-scale dynamics to mechanical instabilities. Philos. Trans. R. Soc. A Math. Phys. Eng. Sci..

[33] Schwartz LW, Roy RV, Eley RR, Petrash S (2001). Dewetting patterns in a drying liquid film. J. Colloid Interface Sci..

[34] Gu X, Raghavan D, Douglas JF, Karim A (2002). Hole growth instability in the dewetting of evaporating polymer solution films. J. Polym. Sci. Part B Polym. Phys..

[35] Zhuang J, Ju YS (2015). A combined experimental and numerical modeling study of the deformation and rupture of axisymmetric liquid bridges under coaxial stretching. Langmuir.

[36] Jeong GS (2016). Viscoelastic lithography for fabricating self-organizing soft micro-honeycomb structures with ultra-high aspect ratios. Nat. Commun..

[37] Park D, Lim J, Park JY, Lee SH (2015). Concise review: Stem cell microenvironment on a chip: Current technologies for tissue engineering and stem cell biology. Stem Cells Transl. Med..

[38] Li Y, Zhang Y, Xie S (2020). A lightweight multilayer honeycomb membrane-type acoustic metamaterial. Appl. Acoust..

[39] Deegan RD (1997). Capillary flow as the cause of ring stains from dried liquid drops. Nature.

[40] Poulard C, Damman P (2007). Control of spreading and drying of a polymer solution from Marangoni flows. Europhys. Lett..

[41] Kanhurkar SD, Gandhi PS, Bhattacharya A (2022). Evolution of mesh-like liquid films in multi-port lifted Hele Shaw cells. Chem. Eng. Sci..

