# Supplementary material for: Controlled viscous fingering in volatile fluid towards spontaneous evolution of ordered 3D patterns
Source: Sci Rep. 2023 Jun 30;13:10610. doi: 10.1038/s41598-023-35510-z (PMC10313706; doi:10.1038/s41598-023-35510-z)
Supplement: Supplementary file 1 — Supplementary Information 1. [file 41598_2023_35510_MOESM1_ESM.pdf]

Controlled viscous fingering in volatile fluid towards spontaneous evolution of ordered 3D patterns

Makrand A. Rakshe[1] **and Prasanna S. Gandhi**[1,*]

[1]Suman Mashruwala Advanced Microengineering Laboratory, Department of Mechanical Engineering, Indian Institute of Technology Bombay, 400076, India
[*]gandhi.iitb@gmail.com

Supplementary Movie. 1. Experiment shown in Figure 5.O_1: The supplementary movie shows stable evolution followed by fluid ring retraction; hence the structure is not retained(No retention).
Supplementary Movie. 2. Experiment shown in Figure 6.$B_{1'}$:The supplementary movie shows a sudden bridge breaking.
Supplementary Movie. 3. Experiment shown in Figure 6.$B_{2'}$: The supplementary movie shows localized bridge breaking during the unstable evolution stage.

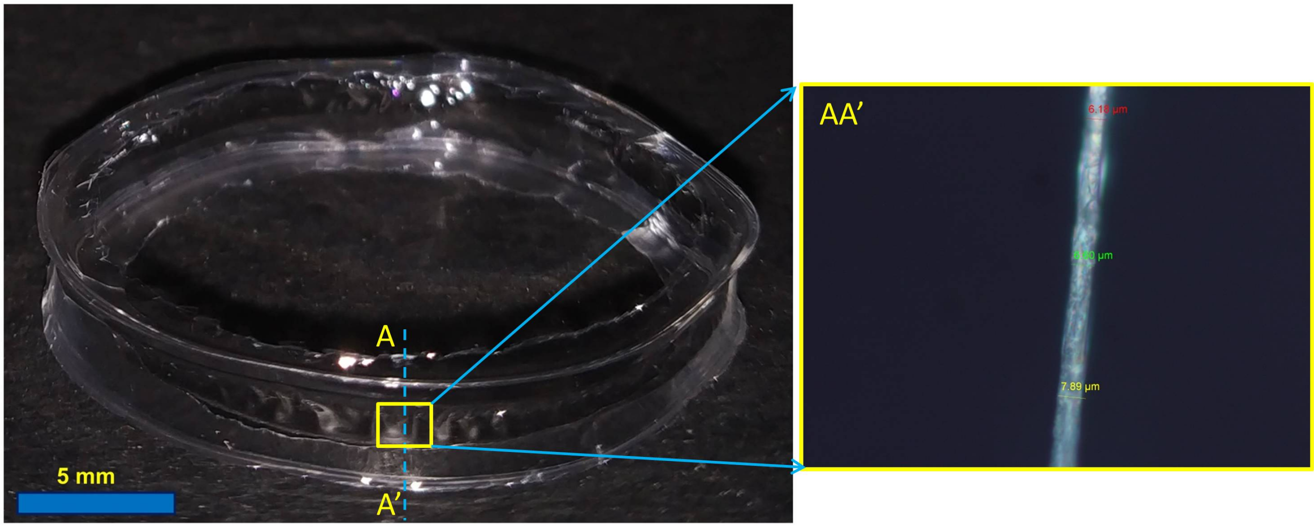

Supplementary Fig. 1. Wall thickness at the mid-plane of the hollow cylindrical well fabricated on ULHSC. Left side image showing a microscopic view of the cross-section(AA’) of the wall at the mid-plane(highlighted section)

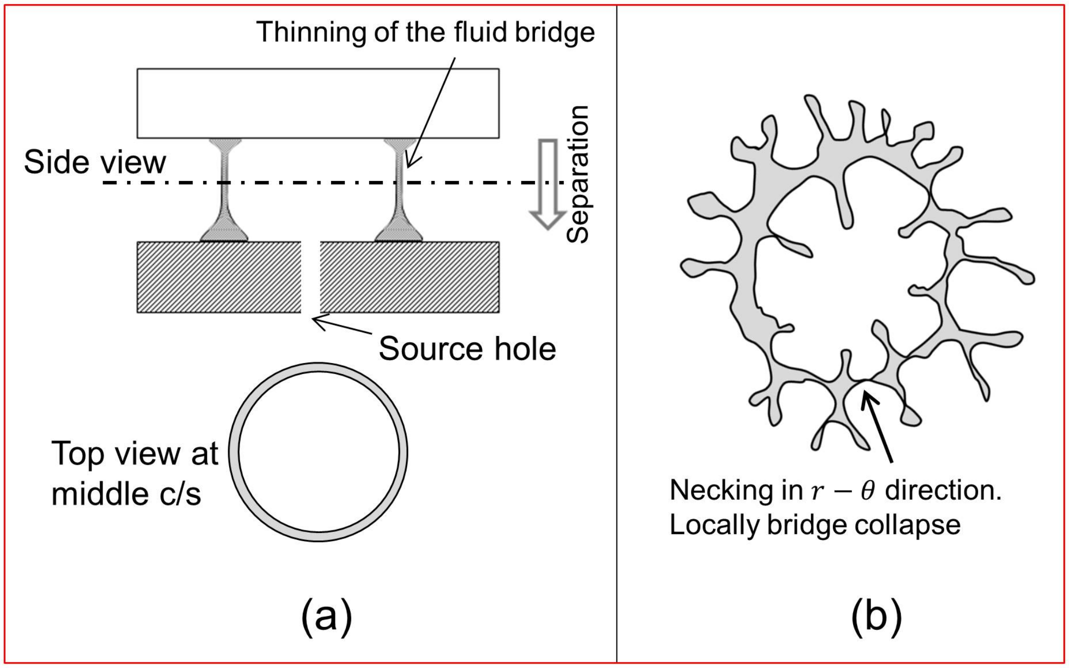

Supplementary Fig. 2. Bridge breaking schematic a) Thining of the fluid bridge during plate separation b) Necking in azimuthal direction during viscous fingering.

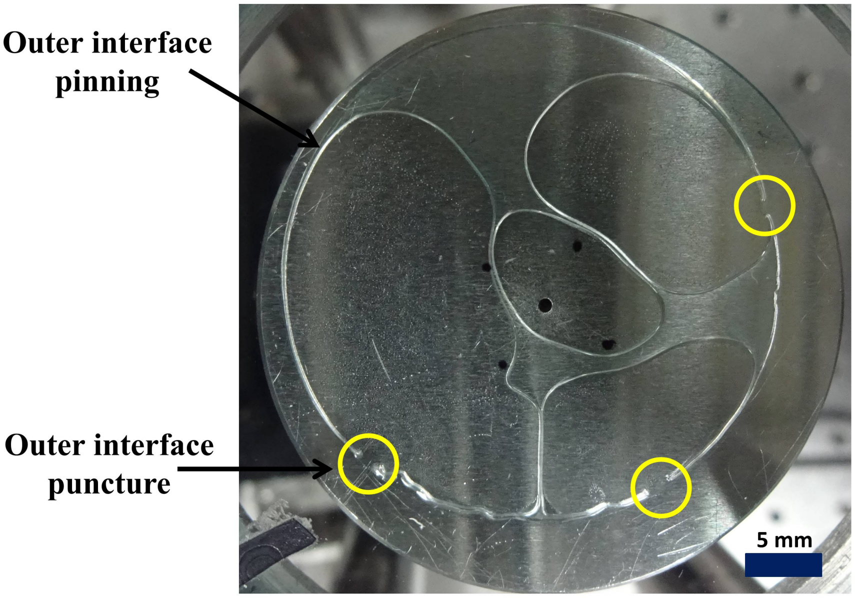

Supplementary Fig. 3. Pinning and Puncture of the outer interface.

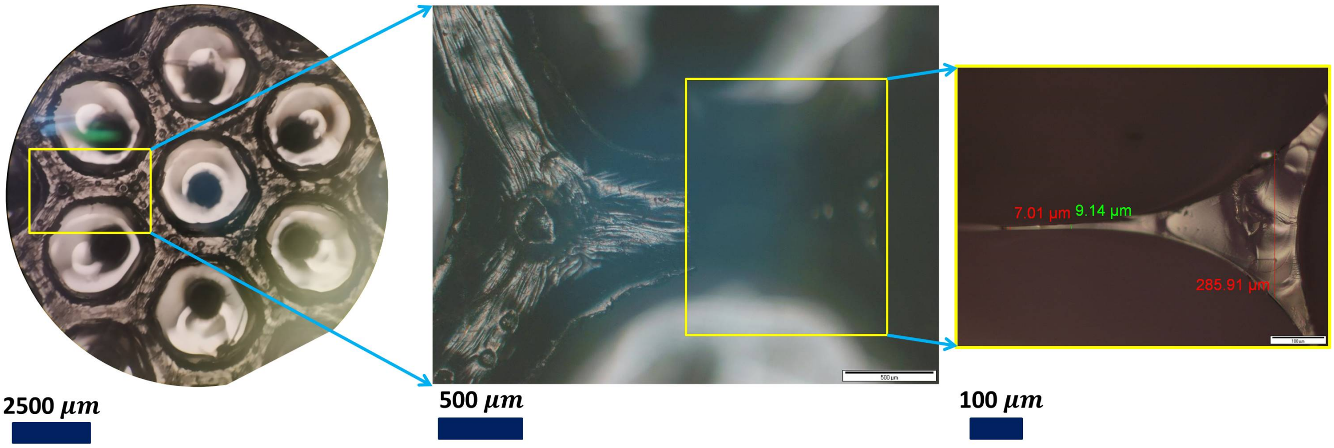

Supplementary Fig. 4. The wall thickness of the mid-plane of the HAR hexagonal mesh fabricated on MLHSC. In the left image, the focus is on the top plane, and in the right image, the focus is on the mid-plane of the highlighted section.

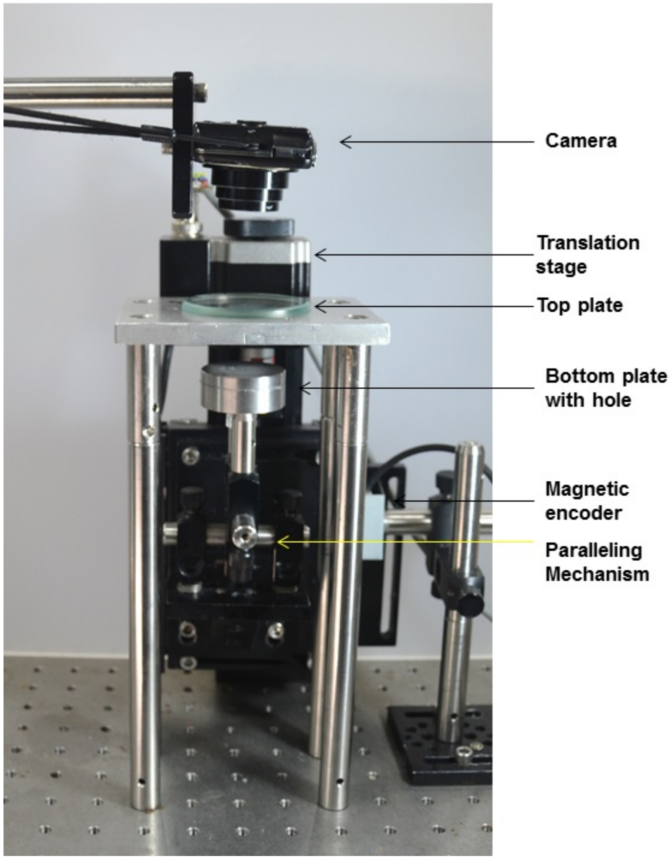

Supplementary Fig. 5. Setup

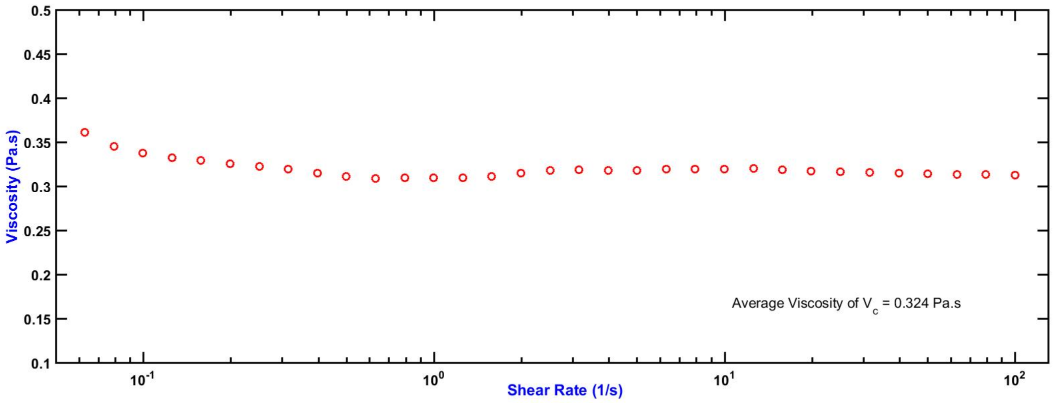

Supplementary Fig. 6. Viscosity of the chloroform solution (V_c)

Evaporation rate (δ) calculation

We took the polymer solution in a cylindrical container (the top side is open to the atmosphere) and kept it on the weighing balance. Then mass loss (in kg/s) is recorded over time and plotted as shown in supplementary Fig. 7. Then evaporation rate δ is calculated as shown below,

Mass flux in kg/m^2s = Evaporation rate in $kg/s \div$ exposing area to atmosphere in m^2

Evaporation rate in m/s = mass flux in $kg/m^2s \div$ density of the fluid in kg/m^3

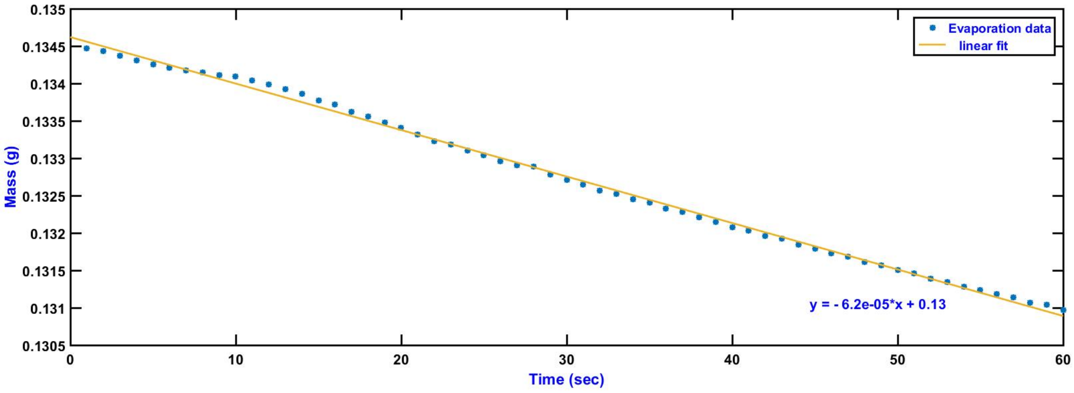

Supplementary Fig. 7. Evaporation rate(g/sec) of the chloroform solution(V_c).

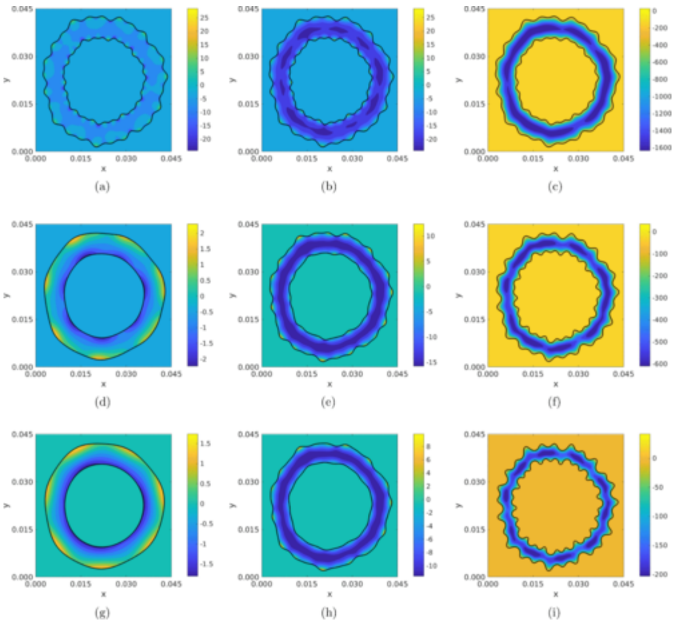

Supplementary Fig. 8. [Figure 18 from Sachin et al., 2019] Air finger evolution on ULHSC for low $\gamma(=1.5)$, (a), (d), (g) for $Ca/(h^*)^3 = 10^0$, (b), (e), (h) for $Ca/(h^*)^3 = 10^2$ and (c), (f), (i) for $Ca/(h^*)^3 = 10^4$. Three time intervals (0 s, 10 s, 24 s) simulation results are shown for different $Ca/(h^*)^3$. Contour shows the pressure distribution in the fluid film where the blue region corresponds to pressure minima.

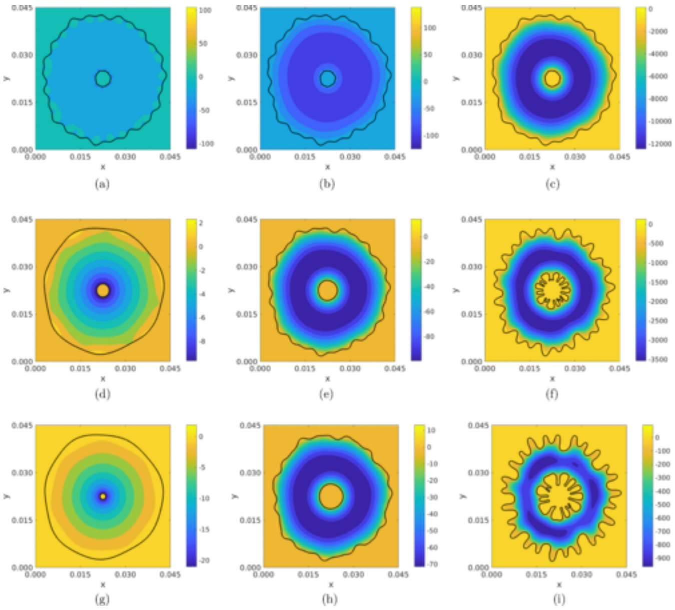

Supplementary Fig. 9. [Figure 19 from Sachin et al., 2019] Air finger evolution on ULHSC for large $\gamma(=8)$, (a), (d), (g) for $Ca/(h^*)^3 = 10^0$, (b), (e), (h) for $Ca/(h^*)^3 = 10^2$ and (c), (f), (i) for $Ca/(h^*)^3 = 10^4$. Three time intervals (0 s, 10 s, 24 s) simulation results are shown for different $Ca/(h^*)^3$. Contour shows the pressure distribution in the fluid film where the blue region corresponds to pressure minima.

Initial pressure profile with high γ (= 45) and high $Ca/(h^*)^3$ (300 for O_1 and 7000 for P_1) would correspond to Figure 19 b and c qualitatively, respectively for O_1 and P_1. Evolution would proceed as in Figure 19 e and 19 h in case of O_1 and as in Figure 19 f and 19 i in case of P_1. With intermediate structures formed in Figures O_1.ii and P_1.ii after retraction of fingers, the new pressure profiles will correspond to Figure 18 g since for both these cases, γ_d is much lower (1.5) now and $Ca/(h_d^*)^3$ also is significantly lower because of the increased gap. At low $Ca/(h^*)^3$, pressure drop within the fluid domain due to lifting

is much lesser than the pressure jump at the interfaces due to the Young-Laplace boundary condition [Sachin et al., 2019]. Thus, pressure from the outer interface to the inner interface reduces monotonically (the pressure gradient at both interfaces is positive), which drives both interfaces radially inward. Finally, the whole fluid is collected in the middle to form a solid cylindrical fluid column, which breaks after further separation.
